# Enhancing Flower Color through Simultaneous Expression of the *B-peru* and *mPAP1* Transcription Factors under Control of a Flower-Specific Promoter

**DOI:** 10.3390/ijms19010309

**Published:** 2018-01-20

**Authors:** Da-Hye Kim, Sangkyu Park, Jong-Yeol Lee, Sun-Hwa Ha, Sun-Hyung Lim

**Affiliations:** 1 National Institute of Agricultural Science, Rural Development Administration, Jeonju 54874, Korea; kimdh143@jbnu.ac.kr (D.-H.K.); psk2779@korea.kr (S.K.P.); jy0820@korea.kr (J.-Y.L.); 2 Department of Genetic Engineering and Graduate School of Biotechnology, Kyung Hee University, Yongin 17104, Korea; sunhwa@khu.ac.kr

**Keywords:** anthocyanin, flower color, flower-specific promoter, transcription factor, tobacco

## Abstract

Flower color is a main target for flower breeding. A transgenic approach for flower color modification requires a transgene and a flower-specific promoter. Here, we expressed the *B-peru* gene encoding a basic helix loop helix (bHLH) transcription factor (TF) together with the *mPAP1* gene encoding an R2R3 MYB TF to enhance flower color in tobacco (*Nicotiana tabacum* L.), using the tobacco *anthocyanidin synthase* (*ANS*) promoter (PANS) to drive flower-specific expression. The transgenic tobacco plants grew normally and produced either dark pink (PANSBP_DP) or dark red (PANSBP_DR) flowers. Quantitative real time polymerase chain reaction (qPCR) revealed that the expression of five structural genes in the flavonoid biosynthetic pathway increased significantly in both PANSBP_DP and PANSBP_DR lines, compared with the non-transformed (NT) control. Interestingly, the expression of two regulatory genes constituting the active MYB-bHLH-WD40 repeat (WDR) (MBW) complex decreased significantly in the PANSBP_DR plants but not in the PANSBP_DP plants. Total flavonol and anthocyanin abundance correlated with flower color, with an increase of 1.6–43.2 fold in the PANSBP_DP plants and 2.0–124.2 fold in the PANSBP_DR plants. Our results indicate that combinatorial expression of *B-peru* and *mPAP1* genes under control of the *ANS* promoter can be a useful strategy for intensifying flower color without growth retardation**.**

## 1. Introduction

Anthocyanins not only contribute to reducing the incidence of major diseases in humans [1], but also are visible pigments conferring orange, red and blue colors in plants [2]. One of the main goals of floral breeding is to develop commercially desirable flower colors through modification and enhancement of anthocyanins. Flower color changes based on genetic manipulation have been reported in a variety of plant species including rose (*Rosa hybrid*), cyclamen (*Cyclamen persicum*) and chrysanthemum (*Chrysanthemum morifolium*) [3,4,5]. Generally, two main strategies are employed to enhance color. One strategy alters the flavonoid-derived pigment profile by overexpressing the gene at the rate-limiting step of the anthocyanin biosynthetic pathway or by blocking the competing biosynthetic pathway. The other approach involves targeting transcription factors that regulate several biosynthetic genes, resulting in the accumulation of flavonoid-derived pigments including anthocyanins, proanthocyanidins, and chalcones.

Anthocyanin biosynthesis is regulated by members of the R2R3 MYB, bHLH, and WDR TF families, which form the MBW complex [6,7]. R2R3 MYBs bind bHLH TFs through a highly conserved N-terminal motif within its DNA-binding domain, and the WDR protein stabilizes the interaction between the bHLH TF and the R2R3 MYB [7,8]. Among the three types of TF that form the MBW complex, only R2R3 MYBs directly bind to the DNA of targets related to anthocyanin biosynthesis. While the active MBW complex promotes anthocyanin biosynthesis, the anthocyanin biosynthesis pathway can be inhibited by inactive MBW complexes involving two distinct types of MYBs: small R3 MYBs and R2R3 MYB repressors [9,10,11]. The small R3 MYBs participate in epidermal cell formation, as well as the regulation of anthocyanin biosynthesis, by interacting with the bHLH TFs in the MBW complex. R2R3 MYBs that act as repressors contain repressive motifs in their C-terminal region, such as the ethylene response factor-associated amphiphilic repression (EAR) motif; these motifs may prevent formation of an active MBW complex for anthocyanin biosynthesis by competing with R2R3 MYB activators for bHLH binding.

To generate new flower colors through enhancing pigment abundance, anthocyanin-related TF genes have been used to alter metabolic flow by funneling precursors toward anthocyanin production. In rose and tobacco, ectopic expression of the Arabidopsis anthocyanin regulator *PAP1*, a R2R3 MYB activator, leads to accumulation of pigment in callus, flowers, and leaves [12,13]. Transgenic Lisianthus harboring the Antirrhinum *ROSEA1* (R2R3 MYB) gene under the control of a constitutive promoter show pigmentation only in sepals [14]. However, transgenic petunia harboring both the *ROSEA1* and *LC* (maize bHLH) genes exhibit distinct color phenotypes in the leaves, anthers, and petal throat regions as well as light-induced intense pigmentation in leaves due to anthocyanin accumulation [14]. Expression of the sweet potato *IbMYB1a* (R2R3 MYB) gene, as well as co-expression of the maize *B-peru* gene with the Arabidopsis *mPAP1* gene, in transgenic tobacco plants results in notable color changes throughout the plant body including callus, seedling, roots, stems, leaves and flowers. These tobacco plants accumulate anthocyanins, but also display a growth-retardation phenotype [15,16,17], suggesting that high accumulation of anthocyanins may have a deleterious effect on plant growth.

Promoters that control gene expression, in a spatially- and/or temporally-specific manner, are needed for successful flower color modification. The promoters of multiple genes involved in pigment synthesis and degradation have an ability to drive such flower-specific expression. The promoters of genes for *carotenoid cleavage dioxygenase 4a-5* (*CCD4a-5*) from chrysanthemum, *chalcone synthase* (*CHS*) from lily (*Lilium oriental*), *anthocyanidin synthase* (*ANS*) from tobacco, and an anthocyanin regulator R2R3 MYB gene (*MYB1*) from Japanese morning glory (*Ipomoea nil*) confer flower-specific activation [18,19,20,21].

In this study, we expressed the anthocyanin regulator genes *B-peru* and *mPAP1* in tobacco under control of PANS, promoter of *ANS* gene from tobacco. Transgenic tobacco plants displayed a darker flower color than control plants. To further investigate this difference in flower color, we examined the expression of anthocyanin biosynthetic pathway genes and the abundance of various flavonoids including flavonols, dihydroflavonols, and anthocyanins. These results illustrate a useful strategy for flower color modification without growth retardation.

## 2. Results

### 2.1. Expression of Anthocyanin Regulators in Floral Tissue of Tobacco Results in Flower Color Changes

**Figure 1:**
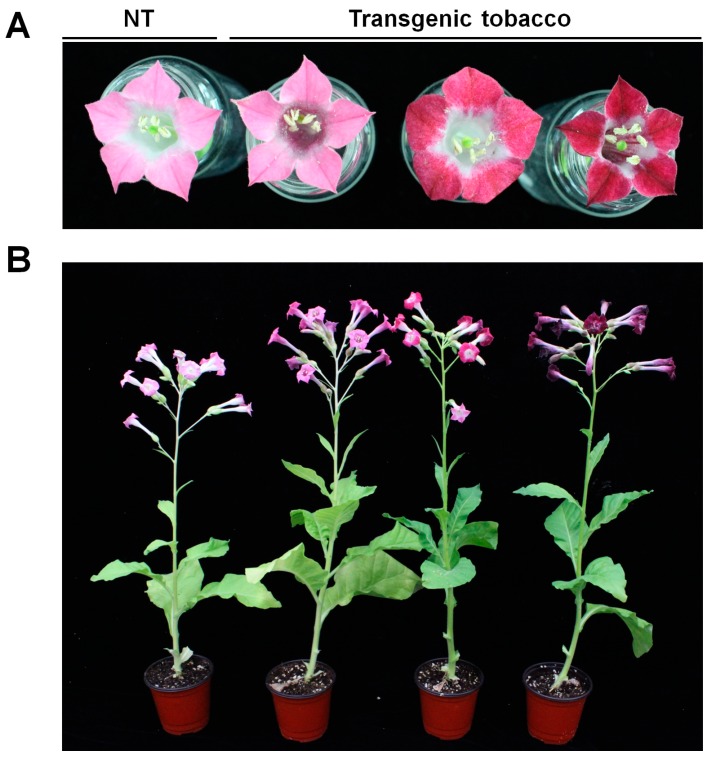
Pigmentation phenotypes of PANSBP transgenic tobacco plants. Representative pigmentation phenotypes of NT and PANSBP transgenic tobacco plants are shown. NT tobacco flowers were pink due to anthocyanin accumulation. The PANSBP transgenic tobacco plants displayed a range of flower colors from dark pink to dark red due to enhanced anthocyanin accumulation in petals (**A**); Leaves lack visible anthocyanin pigmentation in NT and PANSBP transgenic tobacco plants (**B**).

Tobacco is a natural allotetraploid that is derived from the hybridization of *N. sylvestris* and *N. tomentosiformis* [22] and has pink colored flowers. To enhance the pigmentation in tobacco flowers, we constructed the plant expression vector pPANSBP that contains two anthocyanin regulators, *B-peru* of maize and *mPAP1* of Arabidopsis, under control of PANS, a tobacco flower-specific promoter. Twenty independent transgenic (T_0_) PANSBP tobacco plants were obtained using the Agrobacterium-mediated transformation method. The flower color of transgenic plants ranged from dark pink to dark red, as shown in Figure 1A. All of these transgenic T_3_ PANSBP tobacco plants displayed an overall phenotype that was similar to that of NT tobacco plants, except for flower color (Figure 1B).

**Figure 2:**
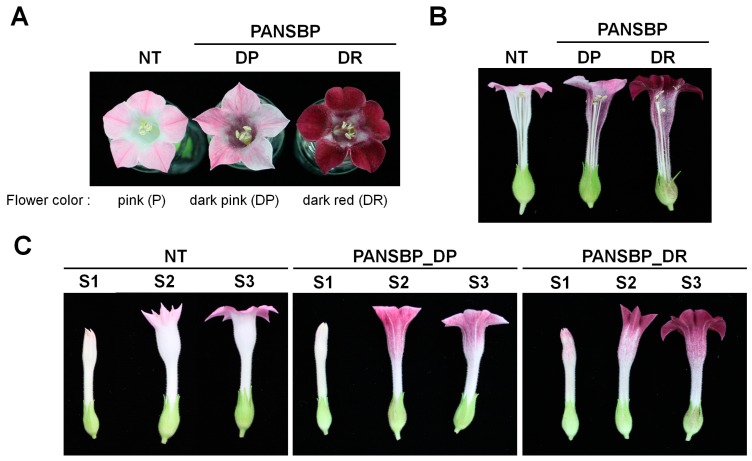
Flower color phenotype of PANSBP transgenic tobacco plants. (**A**) Color comparison of mature flowers from two PANSBP transgenic lines and NT tobacco plants; (**B**) Flowers were hand-sectioned longitudinally to display internal components; (**C**) Flower color variation among phenotypes throughout the three flower developmental stages, S1 to S3.

We selected representative lines for dark pink (PANSBP_DP) and dark red (PANSBP_DR) flowered plants for further study (Figure 2A). NT plants showed the pink color due to anthocyanin accumulation that was present specifically in the corolla limbs, but not in tube of the petal and not in any floral tissues (Figure 2B). PANSBP transgenic plants exhibited the higher anthocyanin accumulation in the petals including corolla limbs and tubes, as well as a slight pigmentation in stamen and sepal, but not in the pistil. We investigated the timing of floral color development in the transgenic plants by assessing pigmentation during three flower developmental stages, stage 1 (S1), S2, and S3. The pink petal color in NT plants became visible at S2 and deepened at S3 (Figure 2C). Petals from the PANSBP_DP line started to show a dark pink color at S2 and maintained it through S3, while PANSBP_DR petals started to show a red pigmentation at S1 and continued to deepen in color until S3.

### 2.2. Measurement of Flower Color and Petal pH

To evaluate these differences in petal colors, colors were measured and expressed on the L*a*b* color coordinate. The L* parameter represents the luminance of the color ranging from 0 (black) to 100 (white). The a* value represents the position between red (positive values) and green (negative values). The b* parameter represents the position between yellow (positive values) and blue (negative values). Additionally, the chroma (C*) of the color represents the saturation, such that a color with a high chroma value looks luminous and concentrated, while the same color with a low chroma value looks dull or grayish. As expected based on appearances, NT plants had the highest L*, the lowest a*, the lowest b*, and the lowest C*, whereas PANSBP_DR plants had the lowest L*, the highest a*, the highest b*, and the highest C* (Table 1). PANSBP_DP plants had the median values for L*, a*, and C*. The colors obtained from the calculated L*, a*, b* and C* coordinates agreed with our visual observation of flower color.

Several studies report that pH is an important factor affecting anthocyanin accumulation [23,24,25,26]. To investigate the relationship between pH and anthocyanin accumulation, we measured the pH of petal cell sap. The petals from PANSBP_DP and PANSBP_DR plants had lower pH values than the petals from NT plants. This result indicates that the lower pH of the cell sap is associated with a darker color in the petals.

### 2.3. Gene Expression of B-peru and mPAP1 under Control of the Tobacco ANS Promoter Was Restricted in Floral Organs 

**Figure 3:**
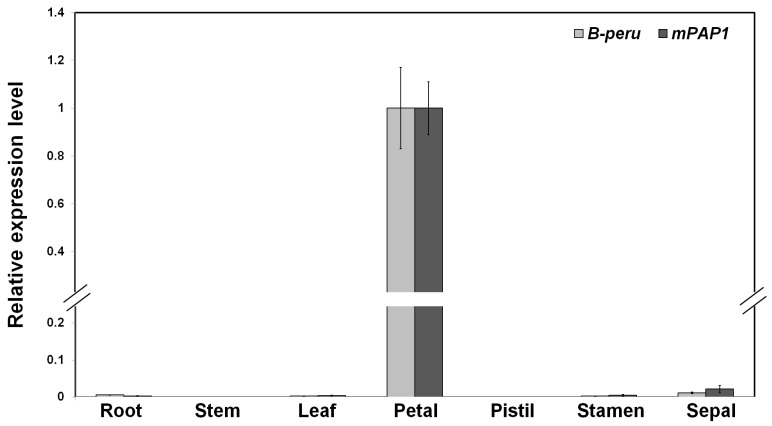
Expression of *B-peru* and *mPAP1* in transgenic tobacco plants transformed with PANSBP. qPCR analysis of the expression levels of *B-peru* and *mPAP1* transgenes in various different tissues of PANSBP-DR line. All results represent mean values ± SD from three biological replicates.

To confirm the tissue-specific expression of *B-peru* and *mPAP1* under control of PANS, we performed qPCR with roots, stems, leaves, petals, sepals, stamens, and pistils. As expected, the expression of the heterologous genes *B-peru* and *mPAP1* was not detected in any tissue from NT plants. In transgenic PANSBP_DR plants, the petals had the highest expression of both *B-peru* and *mPAP1*, followed by the sepals (Figure 3). The expression of *B-peru* was approximately 88 times higher in petals than in sepals and expression of *mPAP1* was approximately 46 times higher in petals than in sepals. Expression of *B-peru* and *mPAP1* was barely detected in the roots, stems, leaves, pistils, and stamens. This result indicates that PANS can restrict gene expression to floral tissues.

### 2.4. Flower Color Modification by Two Anthocyanin Regulators 

**Figure 4:**
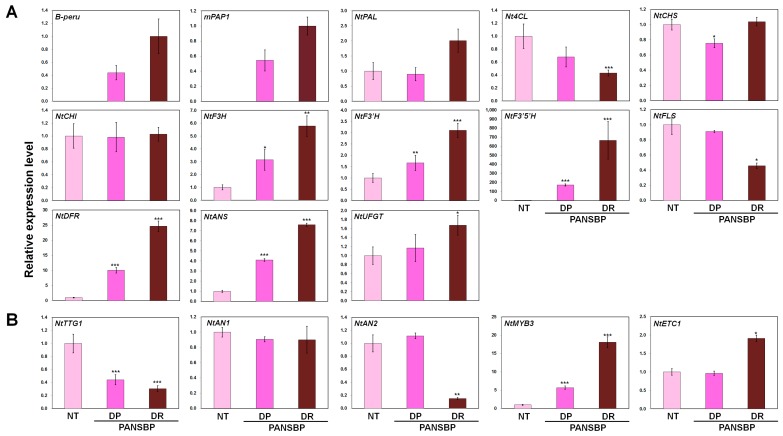
Expression profiles of anthocyanin structural and regulatory genes in NT and PANSBP transgenic tobacco petals. (**A**) Transcript abundance of the *B-peru* and *mPAP1* transgenes and anthocyanin structural genes. The biosynthetic pathway genes evaluated include those encoding *chalcone synthase* (*NtCHS*), *chalcone isomerase* (*NtCHI*), *flavanone 3-hydroxylase* (*NtF3H*), *flavonoid 3′-hydroxylase* (*NtF3′H*), *flavonoid 3′5′-hydroxylase* (*NtF3′5′H*), *flavonol synthase* (*NtFLS*), *dihydroflavonol 4-reductase* (*NtDFR*), *anthocyanidin synthase* (*NtANS*), and *UDP-glucose: flavonoid 3-O-glucosyltransferase* (*NtUFGT*) as well as the upstream enzyme *phenylalanine ammonia-lyase* (*NtPAL*) and *4-coumarate-CoA ligase* (*Nt4CL*); (**B**) T ranscript abundances of endogenous anthocyanin regulators. The tobacco *glyceraldehyde 3-phosphate dehydrogenase* (*GAPDH*) gene was used to normalize the total amounts of RNA for each assay. Bar colors are indicative of flower color phenotypes, pink represents NT; dark pink, PANSBP_DP; dark red, PANSBP_DR. All results represent mean values ± SD from three biological replicates. *, ** and *** indicate values that differ significantly from NT at *p* < 0.05, *p* < 0.01, and *p* < 0.001, respectively, according to a Student’s paired *t*-test.

To further confirm the effect of anthocyanin regulators on flower color, we performed qPCR on petals at S3. As expected, *B-peru* and *mPAP1* transcripts were present in all PANSBP tobacco flowers, and both were more abundant in PANSBP_DR than in PANSBP_DP. To characterize the relationship between color phenotype and transcript level of anthocyanin biosynthetic pathway genes, we measured the expression of eleven structural genes, including the upstream genes *phenylalanine ammonia-lyase* (*NtPAL*) and *4-coumaroyl CoA-ligase* (*Nt4CL*); the early biosynthetic genes (EBGs) *chalcone synthase* (*NtCHS*), *chalcone isomerase* (*NtCHI*), *flavanone 3-hydroxylase* (*NtF3H*), *flavonid 3′-hydroxylase* (*NtF3′H*), *flavonid 3′5′-hydroxylase* (*NtF3′5′H*) and *flavonol synthase* (*NtFLS*); the late biosynthetic genes (LBGs) *dihydroflavonol 4-reductase* (*NtDFR*), *anthocyanidin synthase* (*NtANS*), and *UDP-glucose:flavonoid 3-O-glucosyltransferase* (*NtUFGT*). The schematic diagram of putative flavonoid biosynthetic pathway in tobacco including the above structural genes was provided as Figure S1. In addition, the expression of regulatory genes WDR-type NtTTG1, bHLH activator *NtAN1*, R2R3-MYB activator *NtAN2*, R2R3-MYB repressor *NtMYB3*, and R3 repressor *NtETC1* were analyzed. The expression of five structural genes (*NtF3H*, *NtF3′H*, *NtF3′5′H*, *NtDFR*, and *NtANS*) was higher in all PANSBP tobacco plants compared with NT plants. Transcript levels in PANSBP_DR were higher than those in PANSBP_DP. The transcript levels of *Nt4CL* and *NtFLS* decreased in all transgenic tobacco lines compared with NT plants, although this reduction was more drastic in the PANSBP_DR than in PANSBP_DP. The expression pattern of these increased genes was similar to that of the *B-peru* and *mPAP1* transgenes, whereas the expression pattern of the reduced genes was the opposite of those observed for *B-peru* and *mPAP1* transgenes (Figure 4A). Among the regulatory genes, the *NtTTG1* transcript level was significantly lower in both PANSBP_DR and PANSBP_DP, while the *NtAN1* transcript level did not appear to be affected by the heterologous expression of *B-peru* and *mPAP1* genes. Additionally, the expression of the R2R3-MYB type gene *NtAN2* was significantly reduced but only in PANSBP_DR. Transcript levels for the R2R3-MYB type repressor *NtMYB3* and R3-MYB type repressor *NtETC1* were higher in PANSBP_DR transgenic lines (Figure 4B).

Overall, our expression analysis indicated that the NtANSBP construct was effective at increasing the expression of three EBGs (*NtF3H*, *NtF3′H* and *NtF3′5′H*) and the two LBGs (*NtDFR* and *NtANS*) as well as having a positive regulatory effect on the anthocyanin biosynthetic pathway at the level of transcription in tobacco flowers. These results indicated that heterologous expression of *B-peru* and *mPAP1* genes can modulate the endogenous transcript levels of components of the MBW complex.

### 2.5. Quercetin and Cyanidin Are Significantly Increased in Transgenic Tobacco Flowers 

**Figure 5:**
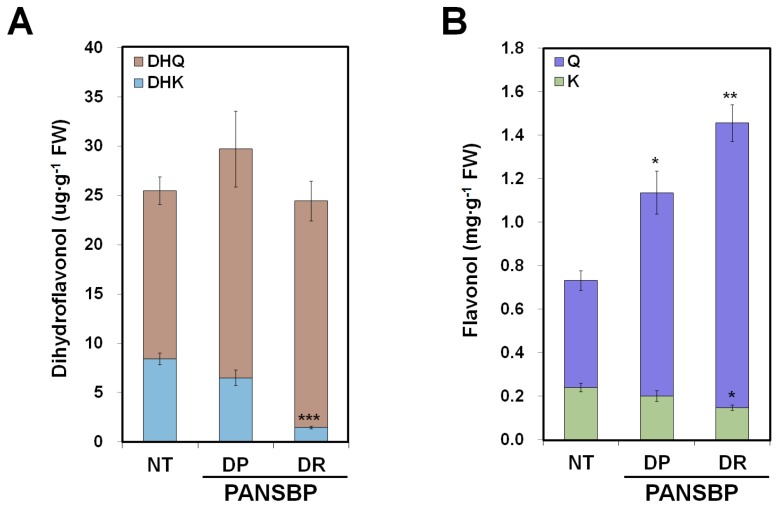
Analysis of dihydroflavonol and flavonol levels in the petals of non-transformed (NT) and PANSBP transgenic tobacco. (**A**) Dihydroflavonol levels in NT and transgenic lines (DHQ, dihydroquercetin; DHK, dihydrokaempferol); (**B**) Flavonols levels in NT and transgenic lines (Q, quercetin; K, kaempferol). All results represent mean values ± SD from three biological replicates. *, ** and *** indicate values that differ significantly from NT at *p* < 0.05, *p* < 0.01 and at *p* < 0.001, respectively, according to a Student’s paired *t*-test.

The total amount of flavonoid aglycones was measured in the flowers of transgenic and NT plants, by separating acid hydrolyzed extracts with high performance liquid chromatography (HPLC) (Figure 5). The peak areas were measured at 288 nm for dihydroflavonols, 350 nm for flavonols, and 520 nm for anthocyanins. Peaks corresponding to the flavanone and flavone standards were not observed at each specific wavelength. The total amounts of dihydroflavonols were comparable in flowers from transgenic plants and NT plants. However, an increase in dihydroquercetin (DHQ) levels and a decrease in dihydrokaempferol (DHK) levels was observed in the flowers of both transgenic lines. The amounts of DHQ in PANSBP_DP (23.22 ± 3.84 µg∙g^−1^) and PANSBP_DR (22.99 ± 2.01 µg∙g^−1^) were slightly higher than that in NT plant (17.06 ± 1.41 µg∙g^−1^), but the amount of DHK in PANSBP_DR (1.46 ± 0.13 µg∙g^−1^) was significantly lower than that in NT plant (8.42 ± 0.60 µg∙g^−1^) (Figure 5A). Large amounts of flavonol aglycones were detected in all PANSBP flowers compared to NT flowers. Total flavonol levels were 0.73 ± 0.07 mg∙g^−1^ in the flowers of NT plants and were 1.6-fold and 2.0-fold higher in PANSBP_DP (1.14 ± 0.12 mg∙g^−1^) and PANSBP_DR (1.46 ± 0.10 mg∙g^−1^) lines, respectively. Furthermore, the flavonol level in PANSBP_DR plants was 1.28 fold higher than that in PANSBP_DP plants. Similar to the accumulation pattern for dihydroflavonols, an increase in quercetin (Q) levels and a decrease in kaempferol (K) levels was also observed in both transgenic lines (Figure 5B).

**Figure 6:**
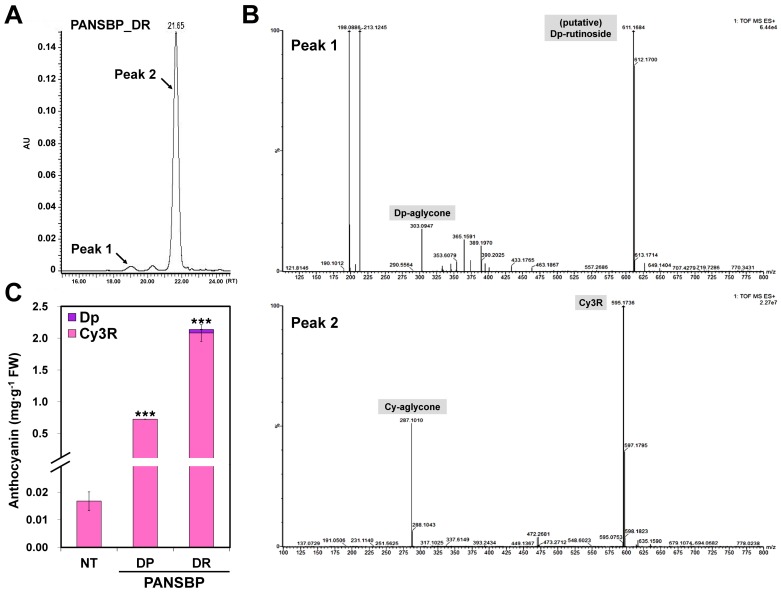
Analysis of anthocyanin levels in the petals of non-transformed (NT) and PANSBP transgenic lines. (**A**) UPLC chromatogram of anthocyanins extracted from the petals of PANSBP_DR line; (**B**) TOF (quadrupole time of flight) mass spectra of the peak 1 and peak 2; (**C**) Anthocyanin levels in NT and transgenic lines (Dp, delphinidin derivative; Cy3R, cyanidin-3-*O*-rutinoside). The result of (**C**) represents mean values ± SD from three biological replicates. *** Indicate values that differ significantly from NT at *p* < 0.001 according to a Student’s paired *t*-test.

To identify the individual anthocyanin glycosides, unhydrolyzed extracts from flowers of NT plants and the transgenic lines were subjected to ultra-performance liquid chromatography-diode array detector-electrospray ionization-quadrupole time of flight mass spectrometry (UPLC-DAD-ESI-QTOF/MS) analysis. The major anthocyanin in the pink flowers of tobacco is known to be cyanidin-3-*O*-rutinoside (Cy3R) [27]. Our results also showed the major UPLC peak at 520 nm corresponding to Cy3R showing a *m*/*z* 595 [M]^+^ in the tobacco petals. Moreover, the trace level of delphinidin derivative showing a *m*/*z* 611 [M]^+^ that can be assumed to be delphinidin-3-*O*-rutinoside was detected in the PANSBP-DR line (Figure 6A,B). The amounts of Cy3R and delphinidin derivative were tentatively determined by applying cyanidin-chloride standard area. The amounts of Cy3R were 0.72 ± 0.003 mg∙g^−1^ and 2.08 ± 0.13 mg∙g^−1^ in PANSBP_DP and PANSBP_DR lines, respectively, which was 43.2 fold and 124.2 fold higher, respectively, than those of NT tobacco (16.76 ± 3.36 µg∙g^−1^) and the level of delphinidin derivative in PANSBP_DR lines was 0.06 ± 0.13 mg∙g^−1^ (Figure 6C). Similar to the accumulation pattern observed for flavonol aglycones, the level of total anthocyanins was approximately 2.9 fold higher in the PANSBP_DR line than in the PANSBP_DP line. These results show that the PANSBP transgenic lines accumulate high levels of flavonoids derived from Qand cyanidin, and clearly demonstrate that the enhanced red pigmentations in the petals of transgenic lines can be attributed to the simultaneous expression of *B-peru* and *mPAP1*.

## 3. Discussion

Anthocyanins accumulate in many flowers, where they are responsible for the orange, red and blue colors and therefore, exert an influence on flower aesthetics. Several studies have reported the relationship between anthocyanin content and cell sap pH in petals [23,24,25,26]. Enhancing the synthesis of cyanidin derivatives through ectopic expression of grape *VvMYB5a*, *VvMYB5b* and *VvMYBA1* genes reduces the pH in petals from transgenic petunia [25]. Accordingly, a loss of anthocyanin pigment due to a mutation in *ANTHOCYANIN1* (*AN1*), *AN2*, and *AN11* in petunia increases the pH in petal extracts [26]. It was also reported that an acidic pH in rose, petunia, and morning glory petals has a positive effect on the stabilization of anthocyanin pigments [24,28,29]. Similarly, in this study we observed an increase in total anthocyanin content and a decrease in pH of the petals upon ectopic expression of two heterologous anthocyanin regulators, *B-peru* and *mPAP1* (Table 1). 

Ectopic expression of anthocyanin biosynthetic regulators of transgenic plants has been reported to lead to visible pigment accumulation in the leaves, stems, flowers and roots [15,16,29]. In some cases, high accumulation of anthocyanins in transgenic tobacco has had a negative effect on plant growth and seed development [15,16]. In this study, the expression of two anthocyanin regulators under the control of a flower-specific promoter, PANS, led to a higher anthocyanin accumulation in floral organs specifically, while displaying a similar growth phenotype to the NT plants (Figure 1). These results suggest that the PANS promoter can be useful for changing flower color or modifying floral morphology without affecting plant growth.

Previous studies on anthocyanin-producing tissues from various plants, show that when the expression of EBGs and LBGs is up-regulated, carbon flux is directed towards anthocyanin biosynthesis [30,31,32]. Similarly, we found that the increased expression of the structural genes, including *NtF3H*, *NtF3′H*, *NtF3′5′H*, *NtDFR* and *NtANS* (Figure 4), leads to large accumulations of flavonoid-derived metabolites affecting the flower color in PANSBP transgenic lines (Figure 5). Specifically, the abundance of flavonols increased approximately 1.6–2 fold, and the abundance of cyanidin derivatives increased approximately 43.2–124.2 fold in the PANSBP transgenic lines. These results indicate that the increment in the expression of the structural genes through the simultaneous expression of *B-peru* and *mPAP1* led to the overall increase in metabolic flux towards flavonoids biosynthesis.

The expression level of *NtF3′H* increased significantly in the PANSBP transgenic lines (Figure 4), which was associated with an increase in the abundance of DHQ and Q, and a decrease in the abundance of DHK and K (Figure 5). The almost exclusive occurrence of cyanidin derivatives in tobacco flowers (Figure 6) also indicates that *NtF3′H* plays a pivotal role in determining the specific pattern of flavonoid accumulation in tobacco flowers.

Until recently, it has been known that delphinidin-derived anthocyanins are not produced in the tobacco petals through the intrinsic biosynthetic pathway, suggesting the absence of *F3′5′H* activity in tobacco. However, a putative *NtF3′5′H* gene sequence has been reported in the recently published draft genome of *N. tabacum* [22]. Interestingly, a small amount of delphinidin was detected in the PANSBP_DR line in this study (Figure 6), which strongly suggests that delphinidin biosynthesis is possible in tobacco petals due to *NtF3′5′H* activity. In this case, the dramatically increased expression of *NtF3′5′H* in the PANSBP transgenic lines can be assumed to be induced by overexpression of *B-peru* and *mPAP1* (Figure 4A). To elucidate the mechanism of production of delphinidin derivatives in tobacco, the spatial and temporal expression pattern and enzymatic characteristics of *NtF3′5′H* should be intensively studied.

The drastic increase in the abundance of cyanidin derivatives in the PANSBP transgenic lines seems to be a result of the significant increase in the expression of LBGs through simultaneous expression of both *B-peru* and *mPAP1* genes. *DFR* is a key enzyme in anthocyanin biosynthesis competing with FLS for the common substrates, dihydroflavonols. The petals of tobaccos transformed with petunia, rose and peach *DFR* genes showed deep red colors due to anthocyanin accumulation, in which the expression levels of *NtFLS* were lowered [33]. Moreover, overexpression of *FLS* genes in tobacco led to down-regulated expression of *NtDFR* and resulted in white colored flower due to predominant accumulation flavonols [33]. The expression pattern of *DFR* and *FLS* is tightly associated the accumulation of anthocyanins and flavonols, resulting in red and white colored flower. As with previous reports, our results indicate that expression profile between *DFR* and *FLS* genes in the PANSBP transgenic lines contributes the increased the metabolic flux toward anthocyanin biosynthesis.

DFR catalyzes the NADPH-dependent reduction of dihydroflavonols into leucoanthocyanidins. Although DFRs show broad substrate specificity for the three dihydroflavonols (DHK, DHQ and dihydromyricetin), some DFRs exhibit different substrate specificity, resulting in species-specific anthocyanin pigments accumulation. Petunia’s DFR does not prefer DHK as a substrate and thus produces almost no pelargonidin-based anthocyanin [34], while DFRs from *Rosa chinensis* and *Calibrachoa hybrida* effectively reduce DHK [35]. In the case of NtDFR, its substrate specificity is still unclear. In the tobacco petals, 4′- and 3′,4′-dihydroxylated forms of dihydroflavonols and flavonols coexist, while only 3′,4′-dihydroxylated form of anthocyanin (cyanidin derivatives) occurred (Figure 5 and Figure 6). It is presumed that this is due to the substrate specificity of NtDFR. Another possibility is that metabolon formation through the physical interaction of NtDFR and *NtF3′H* could contribute to the exclusive accumulation of cyanidin derivatives. This hypothesis seems to be plausible considering the previous report suggesting that CHS, F3H, F3′H, DFR, and ANS of rice could interact with each other to form metabolon for flavonoid biosynthesis [36]. 

Interestingly, the transcript levels of endogenous regulators of the MBW complex were significantly altered in the PANSBP transgenic line. Several studies have reported that the transcript level of *TTG1*, which encodes a WDR protein, displays a similar expression pattern between anthocyanin-accumulating and acyanic tissues [30,37]. However, we found that the transcript levels of endogenous *NtTTG1* decreased significantly in both the PANSBP_DP and PANSBP_DR transgenic lines, the petals of which displayed large amounts of anthocyanin (Figure 5C). Also, the expression of the *NtAN2* gene, which are key regulators of R2R3 MYB activator genes in anthocyanin biosynthesis, was significantly reduced in the PANSBP_DR transgenic line. In addition, we showed that not only R2R3 MYB repressor *NtMYB3*, but also the R3 MYB repressor *NtETC1* increased in expression in the PANSBP transgenic lines. These results indicate that excess anthocyanin accumulation can regulate gene expression through feedback inhibition. Furthermore, the excess of anthocyanin may induce the expression of negative regulators such as *NtMYB3* and *NtETC1*, to control the expression of the MBW complex and fine-tune metabolic flux. This possibility warrants further investigation in tobacco, as do the detailed mechanisms of regulation for the components of the MBW complex as they relate to anthocyanin biosynthesis.

Taken together, these results suggest that simultaneous heterologous expression of *B-peru* and *mPAP1* under PANS can be used to modify anthocyanin content specifically in flowers to intensify flower color without any deleterious effect on growth.

## 4. Materials and Methods

### 4.1. Gene Isolation and Vector Construction

The *mPAP1* and *B-peru* genes were amplified with PCR from cDNA that was prepared from Arabidopsis and maize leaves, respectively, as previously described [17]. Amplified DNA products were cloned into the Gateway entry vector pDONR221 (Invitrogen, Carlsbad, CA, USA) through PCR with the *att* recombination adapter primers according to the manufacturer’s instructions, and clones were verified by DNA sequencing. Subsequently, the *mPAP1* and *B-peru* genes were inserted into the destination vector pB7WG2D, in which the CaMV 35S promoter drives gene expression [38] and contains the Bar gene encoding phosphinothricin acetyltransferase as a selectable marker. The resulting vectors were designated pPB and pBB, respectively. 

To isolate a flower-specific promoter region, PANS was generated by PCR with tobacco genomic DNA as previously described [20]. We generated the recombinant construct PANS::mPAP1::T35S by amplifying PANS and mPAP1::T35S fragments with specific primer sets (PANS-F/PANS-R and mPAP1-F/T35S-R, respectively) and then ligating them together. For constructing PANS::B-peru::T35S, a fragment of PANS was amplified with primer set (PANS-*Hin*dIII-F/PANS-*Spe*I-R), digested with *Hin*dIII and *Spe*I, and then ligated into similarly digested pBB. To construct the final vector for flower-specific expression of both genes (pPANSBP), the PANS::mPAP1::T35S fragment was amplified with PANS-*Hin*dIII-F/T35S-R1 primers, and then PANS::B-peru::T35S and the PANS::mPAP1::T35S fragments were digested with *Hin*dIII and ligated together. The resulting vector, pPANSBP (Figure S2), was transferred into *Agrobacterium* strain GV3101 using the freeze–thaw method. All gene-specific primers are listed in Table S1.

### 4.2. Plant Regeneration 

Transgenic tobacco (*N. tabacum* cv. Xanthi) plants were generated by transforming tobacco with Agrobacterium containing the pPANSBP construct with the leaf disc method [39]. Briefly, tobacco seeds were surface sterilized and grown on solidified half-strength Murashige and Skoog (MS) medium. The plants were grown in a growth room under 16 h light/8 h dark cycles at 26 ± 1 °C for two months. Tobacco leaf discs were obtained from the cultured plants and were submerged in the Agrobacterium mixture. To identify transgenic events, explants were cultured on shoot-inducing medium containing 10 mg/L phosphinothricin. Regenerated shoots were subsequently transferred to a root-inducing MS medium containing 10 mg/L phosphinothricin without selection to enable rooting prior to being transplanted into a greenhouse and cultivated to maturity. The transgenic tobacco plants were grown to maturity and seeds were obtained by self-pollination. Two representative transgenic pPANSBP tobacco lines were selected for further analysis. Transgenic T_3_ lines were developed by successive self-pollination of T_0_ plants. The flower phenotypes were checked at three flower developmental stages, S1, S2, and S3.

### 4.3. Measurement of Flower Color and Petal pH

Petal color was measured with lightness (L*) and two chromatic components a* and b* of the CIE L*a*b* color coordinate [40] using a hand colorimeter (CS-210 Precise Colorimeter, CHN Spec Technology, Ltd., Hangzhou, China). L* values indicate lightness (0 = black, 100 = white); the a* and b* values shift from negative to positive values to indicate the shift from green to red and from blue to yellow, respectively. Three areas of the adaxial surface were subjected to color measurements. Scores from three replicate petals at stage 3 from three different flowers were averaged.

Relative changes in cell sap pH (correlating to changes in vacuolar pH) were determined by extracting 0.5 g of macerated petals in 5 mL of bidistilled water. After 2 h of occasional stirring, the pH of the solution was measured with a pH meter (S20 SevenEasy™; Mettler Toledo, Columbus, OH, USA). Each measurement was repeated with five individual flowers for each developmental stage.

### 4.4. Total RNA Extraction and qPCR Analysis 

Total RNA from tobacco roots, stems, leaves, floral tissues including sepals, petals, stamens and pistils were prepared using TRIzol Reagent (Invitrogen) and first-strand cDNAs were generated using the cDNA EcoDry kit (Clontech, Madison, USA). The qPCR conditions and gene-specific primers are described in previous studies [20,30]. Gene-specific primers for *NtF3′5′H*, *NtMYB3* and *NtETC1* are listed in Table S1. *Glyceraldehyde 3-phosphate dehydrogenase* (*GAPDH*) gene, which was previously used in gene expression analysis of transgenic tobaccos was used as an internal reference [8,30]. Three biological replicates were examined for each sample.

### 4.5. Flavonoid Analysis

To analyze flavonoid aglycones, acid hydrolysis was carried out. Flower samples at S3 stage were harvested and disrupted in liquid nitrogen. Then, 50 mg of each sample was mixed with 150 μL of 50% methanol containing 1.2N HCl and incubated at 80 °C for 2 h. After centrifugation at 15,000× *g* for 10 min at 4 °C the supernatant was transferred to a new tube. The remaining pellet was rinsed twice with 50 μL of 50% methanol containing 1.2 N HCl, and each extract was combined and subjected to HPLC. HPLC was performed on an LC-20A HPLC system (Shimadzu, Kyoto, Japan) equipped with an Inertsil-ODS3 C18 column (5 μm, 250 × 4.6 mm, GL Science). The mobile phase was composed of water containing 0.1% formic acid (A) and acetonitrile containing 0.1% formic acid (B). The gradient profile was optimized as follows: 0–30 min, linear gradient 5–55% (*v*/*v*) B; 30–45 min, linear gradient 55–65% (*v*/*v*) B; 45–50 min, linear gradient 65–100% (*v*/*v*) B at a flow rate of 1 mL∙min^−1^. The temperature of the column compartment was maintained at 40 °C. A diode-array detector was used for compound detection. The spectra of the compounds were recorded between 210 and 800 nm and the peak corresponding to each compound was identified by comparing the retention times and UV spectra with those of the standards. 

To identify individual anthocyanin glycosides, UPLC-QTOF/MS analysis was conducted. The ground sample (0.05 g) was mixed with 600 μL of MFW solution (methanol:formic acid:water; 50:5:45; *v*/*v*/*v*) and then incubated at room temperature for 1 h with shaking. After centrifugation at 15,000× *g* for 10 min at 4 °C, the supernatant was transferred to a new tube. Additional extractions with 300 μL of MFW solution for 10 min were repeated twice for each sample, and each extract solution was combined with the first extract. Extracts were filtered through 0.45 μm Teflon polytetrafluoroethylene syringe filters, diluted 10-fold in MFW solution, and then subjected to UPLC-QTOF/MS analysis. The analysis was conducted on UPLC-DAD-ESI-QTOF/MS system (Waters MS Technologies, Manchester, UK). The separation was performed with a Luna Omega 1.6 μm column (C18, 150 × 2.1 mm) (Phenomenex, Torrance, CA, USA), a SecurityGuard^TM^ ULTRA cartridges C18 for 2.1 ID pre-column (Phenomenex), and was operated at a temperature of 35 °C. The mobile phase consisted of 0.5% formic acid in water (A) and 0.5% formic acid in acetonitrile (B) at a flow of 0.3 mL∙min^−1^ using the following gradient program: 0–2 min, isocratic 7% (*v*/*v*) B; 2–24 min, linear gradient 7–15% (*v*/*v*) B; 24–40 min, linear gradient 15–30% (*v*/*v*) B; 40–48 min, linear gradient 30–60% (*v*/*v*) B; 48–50 min, isocratic 60% (*v*/*v*) B; 50–53 min, linear gradient 60–90% (*v*/*v*) B; 53–54 min, isocratic 90% (*v*/*v*) B; 54–55 min, linear gradient 90–7% (*v*/*v*) B; 55–60 min, isocratic 7% (*v*/*v*) B. Specific wavelengths were monitored separately at 288 nm for flavanones and dihydroflavonols, and 350 nm for flavones and flavonols, and 520 nm for anthocyanins. The UPLC was coupled to a Xevo G2-S QTOF-ESI/MS (Waters MS Technologies). The scan range was *m*/*z* 50–800 in positive ion mode. The capillary and sampling cone voltages were 3.5 kV and 40 V, respectively. The desolvation gas was maintained at 1050 L∙h^−1^ at a temperature of 500 °C. The cone gas was maintained at 50 L∙h^−1^ with an ion source temperature of 120 °C. Data acquisition and processing was performed using MassLynx version 4.1 software (Waters MS Technologies).

## Figures and Tables

**Table 1:** Colorimetric parameters of petals at stage S3 of flower development in non-transgenic (NT) and PANSBP transgenic tobacco plants.

Line	Flower Color	L*	a*	b*	C*	pH
NT	Pink	78.15 ± 0.81	21.75 ± 1.04	−18.96 ± 0.17	28.85	5.3 ± 0.01
PANSBP_DP	Dark Pink	73.36 ± 1.71	25.44 ± 1.71	−18.33 ± 0.32	31.35	5.2 ± 0.01
PANSBP_DR	Dark Red	38.05 ± 0.96	42.97 ± 1.02	−3.19 ± 1.41	43.09	5.11 ± 0.02

## Supplementary Materials

Supplementary materials can be found at www.mdpi.com/1422-0067/19/1/309/s1. Figure S1. Schematic representation of anthocyanins biosynthetic pathway in tobacco. Figure S2. Schematic representation of the binary vector PANSBP used for *B-peru* and *mPAP1* expression in flowers. Table S1. PCR primers used in this study.
