# Supplementary material for: Enhancing Flower Color through Simultaneous Expression of the B-peru and mPAP1 Transcription Factors under Control of a Flower-Specific Promoter
Source: Int J Mol Sci. 2018 Jan 20;19(1):309. doi: 10.3390/ijms19010309 (PMC5796253; doi:10.3390/ijms19010309)
Supplement: Supplementary file 1 [file ijms-19-00309-s001.pdf]

Supplementary Materials:

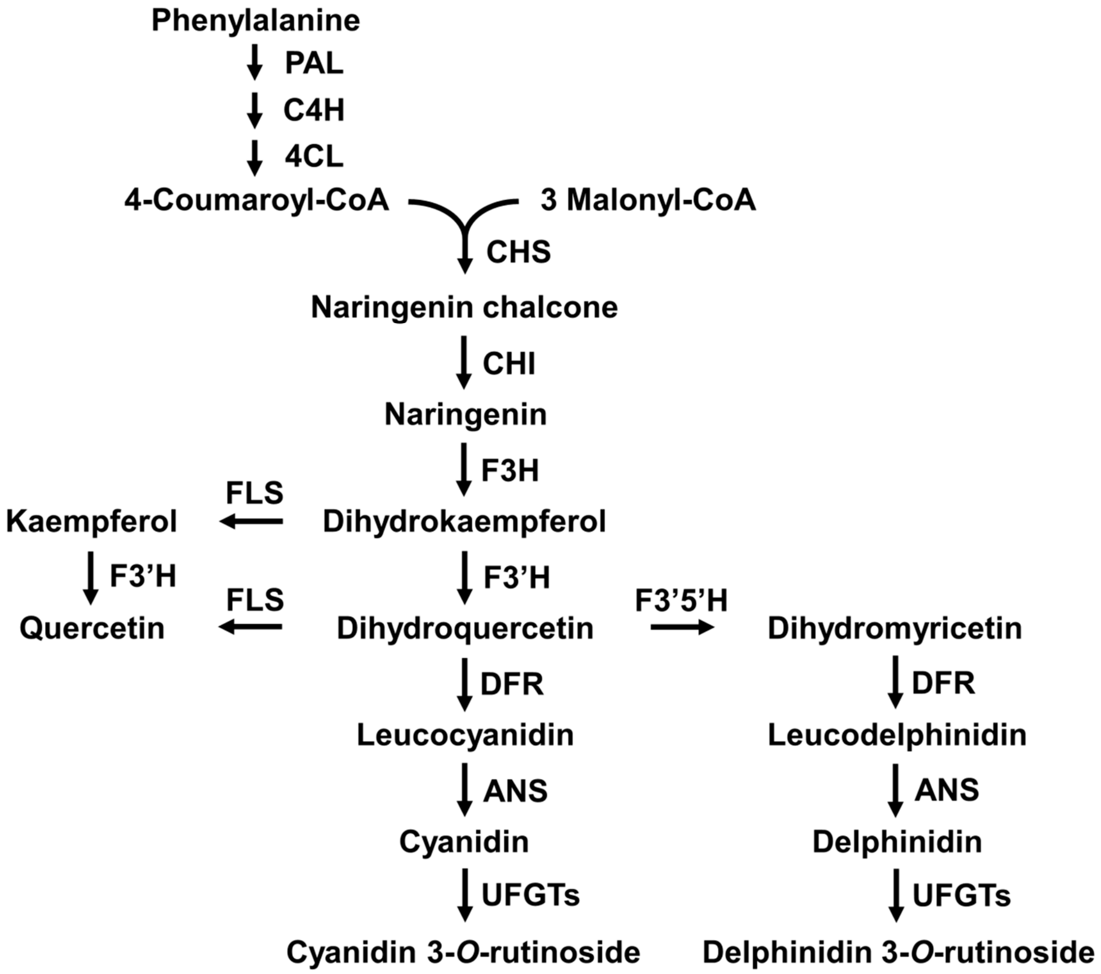

Figure S1. Schematic representation of anthocyanins biosynthetic pathway in tobacco. PAL, phenylalanine ammonia-lyase; C4H, cinnamate 4-hydroxylase; 4CL, 4-coumarate-CoA ligase; CHS, chalcone synthase; CHI, chalcone isomerase; F3H, flavanone 3-hydroxylase; FLS, flavonol synthase; F3′H, flavonoid 3′-hydroxylase; F3′5′H, flavonoid 3′,5′-hydroxylase; DFR, dihydroflavonol 4-reductase; ANS, anthocyanidin synthase; UFGT, UDP-glucose: flavonoid 3-O-glucosyltransferase.

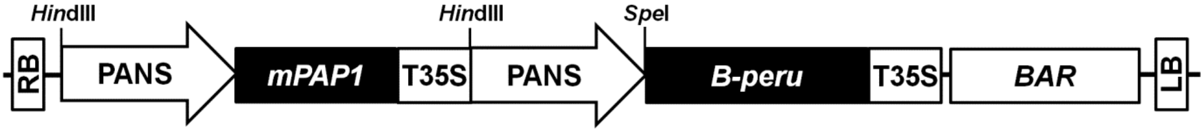

Figure S2. Schematic representation of the binary vector PANSBP used for *B-peru* and *mPAP1* expression in flowers.

Table S1. PCR primers used in this study.

Primer	Sequence	Usage
PANS-F	5′- CCGGTCAACTCAAGACAACCTACCTA-3′	Gene cloning
PANS-R	5′-TTTGTACAAACTTGTCTCTGTATTATTCTT-3′	Gene cloning
mPAP1-F	5′-AAGAATAATACAGAGACAAGTTTGTA CAAA-3′	Gene cloning
T35S-R	5′-TCTAGAGGGCCCGACGTCGCATGCCTGC-3′	Gene cloning
PANS-*Hin*dIII-F	5′-AGCTCAAGCTAAGCTTCCGGTCAACTCAAGACAAC-3′	Gene cloning
PANS-*Spe*I-R	5′-TTGTGATATCACTAGTCTCTGTATTATTCTTGCTTTTTCTT-3′	Gene cloning
T35S-R1	5′-TGAGTTGACCGGAAGCTTTCTAGAGGGCCCGACGTCGCAT-3′	Gene cloning
NtF3′5′H-F	5′-TTGATGTGTGGGAAAAACCA-3′	qPCR
NtF3′5′H-R	5′-CAAAATCGTTCCCTCTTGGA-3′	qPCR
NtMYB3-F	5′-CCGGGGAGAACTGATAATGA-3′	qPCR
NtMYB3-R	5′-TTTCTGGCCAAAACTCAAGG-3′	qPCR
NtETC1-F	5′-TCCTCCTGATTCTCAAGGAAA-3′	qPCR
NtETC1-R	5′-TCCGGGTATTCTTCCAGCTA-3′	qPCR
